# Rapid qualitative analysis approach to stakeholder and client interviews to inform mobile-based HIV testing in the U.S. Deep South

**DOI:** 10.1186/s13690-023-01039-w

**Published:** 2023-02-15

**Authors:** Madeline C. Pratt, Oluwaseyi O. Isehunwa, Ingrid V. Bassett, Mirjam-Colette Kempf, Bretia Gordon, Lynn T. Matthews

**Affiliations:** 1 Division of Infectious Diseases, Department of Medicine, University of Alabama at Birmingham, Ziegler Research Building 210, 1720 2nd Ave S, Birmingham, AL 35294 USA; 2 Division of Infectious Disease and Medical Practice Evaluation Center, Massachusetts General Hospital, Boston, MA USA; 3 Family, Community, and Health Systems, School of Nursing, University of Alabama at Birmingham, Birmingham, AL USA; 4 Departments of Epidemiology and Health Behavior, School of Public Health, University of Alabama at Birmingham, Birmingham, AL USA; 5 Medical Advocacy and Outreach, Montgomery, AL USA

**Keywords:** HIV testing, Rural, Sexual Health, Rapid Qualitative Analysis, Implementation science

## Abstract

**Background:**

The severity of the HIV epidemic in the United States’ rural South highlights geographic, socioeconomic, and racial disparities that disproportionately affect poor Black Americans. Approximately 16% of Alabamians living with HIV remain undiagnosed and just 37% of rural Alabamians have ever been tested for HIV.

**Methods:**

We conducted in-depth interviews with 22 key stakeholders involved in HIV prevention, testing, treatment, or community health initiatives, and 10 adults living in rural communities across Alabama to explore HIV testing challenges and opportunities. We utilized a rapid qualitative analysis approach and engaged community partners for feedback and discussion. This analysis will inform the implementation of a mobile HIV testing service in rural Alabama.

**Results:**

The following themes were identified: (1) Cultural norms, racism, poverty, and rurality impair access to healthcare. (2) Lack of sex education, low knowledge of HIV and perception of risk reinforce stigmas. (3) Messaging about “Undetectable = Untransmissible” (U = U) is not well understood in communities. (4) Community involvement may promote communication and trust between communities and testing advocates. (5) Novel testing strategies are acceptable and may diminish barriers.

**Conclusions:**

Working with community “gatekeepers” may be a key strategy to understand and promote acceptability of interventions new to rural Alabama and ameliorate stigma within communities. The implementation of new HIV testing strategies requires building and maintaining relationships with advocates, especially faith-based leaders, who engage people across many demographics.

**Supplementary Information:**

The online version contains supplementary material available at 10.1186/s13690-023-01039-w.

## Introduction

The rural Deep South of the United States has wide margins of health disparities, and the severity of the HIV epidemic [1] in these states highlights the need to address geographic, socioeconomic, and racial disparities that disproportionately affect poor Black Americans. Southern states accounted for nearly half of incident and prevalent HIV cases in the US in 2018 [2], indicating the need for innovative interventions to increase testing, prevention, linkage to care, and retention. The disparities in health and HIV outcomes are pronounced in Alabama [3–7], where the Black Belt, a region originally named for its rich soil that fueled agricultural practices and where more than half of Alabama’s enslaved population lived in the nineteenth century, is now known for economic disparities and poor access to health care and social services [8]. HIV incidence rates in Alabama have remained approximately the same since 1993, resulting in a stagnation of the HIV epidemic in the state [9, 10]. The U.S. 2019 plan for Ending the HIV Epidemic (EHE) [11] leverages scientific advancements in diagnosis, prevention, treatment, and outbreak response with the goal of reducing new HIV infections in the country by 90% by 2030. Alabama is one of seven EHE focus states characterized by high HIV incidence rates in rural counties.

“Diagnose”, the first of four EHE pillars, is a critical step to curbing the epidemic through widespread testing and early detection [11]. In 2018, the CDC estimated approximately 16% of Alabamians living with HIV remained undiagnosed [12, 13], and only 37% of rural Alabama respondents to the CDC Behavioral Risk Factor Surveillance System (BRFSS) survey indicated ever having been tested for HIV [14]. Testing and diagnosis data from commercial and public sources show a median of 3.9 (IQR 0–9.5) years from infection to diagnosis in Alabama [15], compared to a median of 3.0 (IQR 0.7–7.8) years in the U.S. as a whole [16]. As undiagnosed people living with HIV (PLWH) may contribute to as many as one-third of new HIV infections [17], it is crucial to promote and provide access to HIV testing in rural communities in need of HIV testing.

While data on barriers to and promoters of HIV testing in Alabama specifically are limited, studies exploring HIV testing in the rural South describe that willingness to test is impacted by HIV conspiracy theories, low partner status disclosure, distance to testing sites and transportation issues, lack of time, perceived costs, shortage of health care facilities, and low perceived risk [18–22]. Some promoters of testing may include increased patient-provider engagement, integrating HIV testing with other health services, rapid HIV testing, free testing services, and more accessible testing locations [19, 21, 23]. Qualitative data from perspectives of both clients and stakeholders are more limited, with few studies exploring promoters and barriers of testing and prevention [21, 24, 25]. Data are needed to understand how to improve HIV testing interventions in rural communities and implement novel testing strategies to reach vulnerable populations.

This qualitative study was a part of a larger mixed-methods study [15] aiming to identify key regions and populations lacking HIV testing and understand the feasibility, acceptability, and appropriateness [26] of novel testing interventions in rural Alabama counties. This paper explores barriers to and promoters of HIV testing shared from diverse perspectives, including health department and hospital managers, testing and outreach coordinators, clergy, community leaders, and people living in rural Alabama. These data will inform the implementation of mobile-based HIV counseling and testing (MHCT) and other novel (to this setting) testing strategies through the understanding of challenges, disparities, and barriers to accessing HIV testing in the rural South.

## Methods

### Setting

**Fig. 1:**
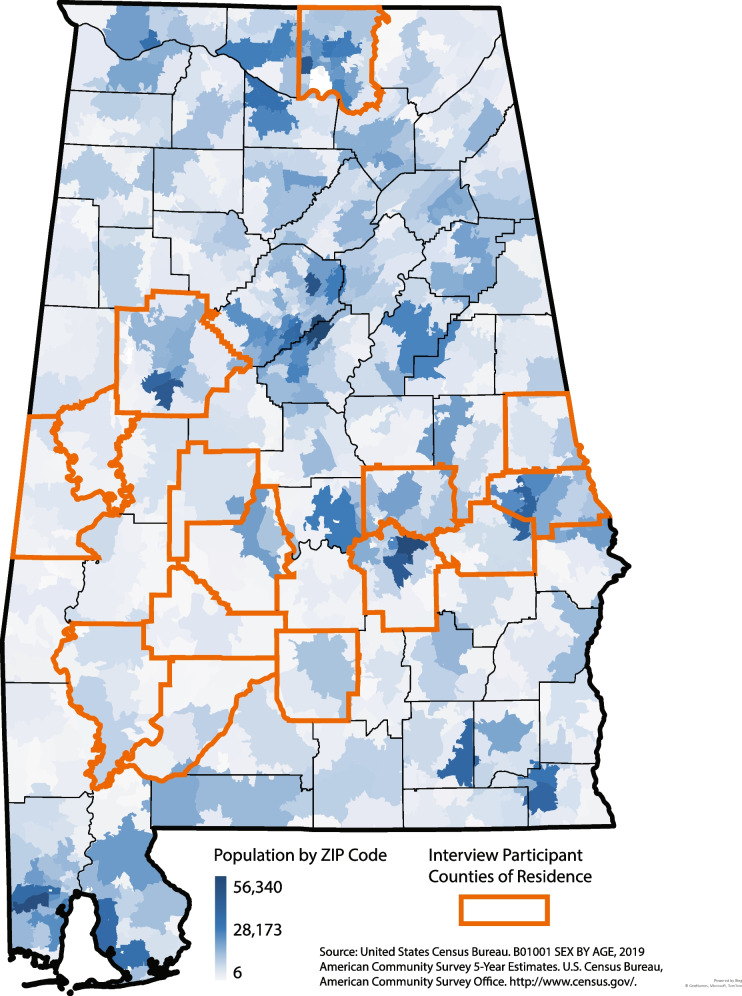
Population of Alabama by zip code and in-depth interview participants’ counties of residence

We recruited participants from counties across Alabama, with purposive sampling from the Black Belt and surrounding rural counties, defined as areas outside of urbanized areas and urban clusters by the United States Census Bureau Office of Management and Budget [27]. Figure 1 shows the population of Alabama by zip code and the counties from which stakeholders were interviewed.

### Participants

#### Stakeholders

Providers, advocates, public health professionals, counselors, community leaders, and community members involved in the promotion or execution of HIV testing, prevention, and/or linkage to treatment, who were at least 18 years of age and able to consent were eligible to participate in an in-depth interview. Participants were recruited via email invitation distributed by our community partners including AIDS Service Organizations in Montgomery (Medical Advocacy and Outreach (MAO)), Tuscaloosa (Five Horizons), and Huntsville (Thrive); the Black Belt Community Foundation (BBCF) in Selma, and the Alabama Department of Public Health (ADPH). Team members also attended virtual meetings to invite potential participants; these included an Alabama HIV Prevention Managers and Community Partners monthly meeting and a quarterly collaboration meeting with MAO, ADPH, and the University of Alabama at Birmingham Center for AIDS Research (UAB CFAR). We worked with the UAB CFAR Ending HIV in Alabama Scientific Working Group to identify community-based organizations and community leaders across Alabama. Snowball sampling was used, as participants were asked if they knew of other community members or advocates involved in HIV testing, prevention, and/or treatment who we could contact.

#### Clients

People over the age of 18, living in Alabama, who had ever or never been tested for HIV, and were able to consent were eligible to participate in an in-depth interview. Participants were recruited through stakeholders, community partners, and through flyers and study staff at a rural health fair.

### Data collection

Separate interview guides were developed for stakeholder and client participants [see Additional Files 1 and 2], using the Framework for Health Communication Across the HIV-Care Continuum [28] to explore intrapersonal, interpersonal, health services, community, and policy factors that impact access to and uptake of HIV testing in rural Alabama. The interview guides were designed to assess the acceptability, feasibility, and appropriateness, as defined by Weiner et al*.* [26], of mobile-based HIV testing and secondary distribution of self-testing kits to inform the future implementation of a mobile-testing van by MAO. Quantitative geospatial data showing HIV testing rates per 100,000 population from this study were integrated into the stakeholder interview guide to assess geographic priorities for testing and outreach, from the perspective of advocates and community leaders across the state.

Demographic data were collected from each participant prior to completing an in-depth interview using UAB’s Qualtrics survey platform.

Stakeholder interviews were conducted between November 2020 and May 2021, and client interviews were conducted between May and August 2021 by a trained research associate. Given COVID-19 pandemic considerations, all interviews were conducted via telephone or HIPAA-compliant Zoom calls. Participants were read the informed consent document and given a chance to ask questions before verbally consenting to participate. The interviews lasted 15—96 min, were audio-recorded, and transcribed.

### Data analysis

Transcripts were reviewed by two team members for accuracy (MCP and LTM). A rapid qualitative analysis approach [29] was used. A domain name was created for each corresponding interview question (e.g., stigma, race, rurality), then one transcript summary template including all identified domains was drafted for the stakeholder interviews and one for the client interviews [see Additional Files 3 and 4]. Under each domain, the summary included sections for main ideas, impactful quotes, intersecting domains, and the applicable determinants of the Consolidated Framework for Implementation Research (CFIR) [30] [see Additional Files 3 and 4]. Both summary templates were piloted with two interviews by the analysis team (MCP, LTM, OOI) to determine usability and relevance. They were revised and piloted several times until the final summary templates were agreed upon and consistency across team summarization was achieved, the transcripts were divided among three team members (MCP, LTM, OOI) for summarization. After the transcripts were summarized, the key points and impactful quotes were compiled into a summary matrix representing all the transcripts, with participants’ coded IDs as rows and the domains as columns (see supplementary materials). Four team members (MCP, LTM, OOI, MCK) discussed the key points and intersections of domains to reduce the matrix to emerging themes.

**Fig. 2:**
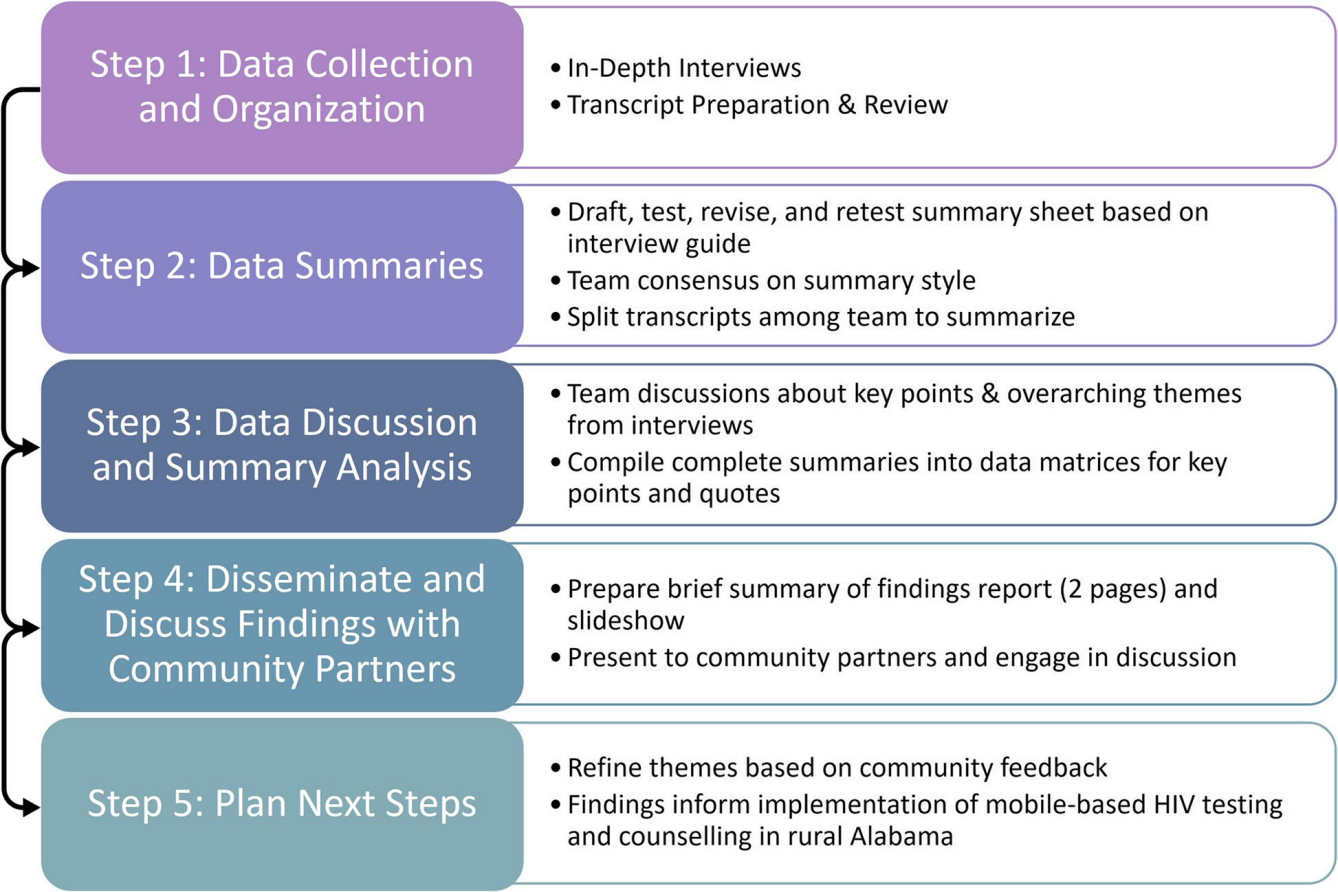
Rapid qualitative analysis approach

The findings were compiled into a summarized two-page report (see supplemental materials) including the following headings: Big Picture Barriers and Promoters, Advantages of MHCT, Disadvantages of MHCT, Community Perceptions of MHCT, Resources and Support Needed for MHCT, Sites for MHCT, HIV Testing Messaging, and Self-Testing Kits and Distribution. These findings were also summarized in a brief slide presentation for leaders from MAO and BBCF at two virtual meetings in July and August 2021. The findings were discussed with MAO education and outreach coordinators in October 2021. Feedback from discussion sessions were recorded, and the primary study team discussed, wrote, and revised the themes presented here based on community feedback. Figure 2 shows the rapid qualitative analysis approach.

### Ethics

This study was approved by the University of Alabama at Birmingham’s Institutional Review Board (Approval # IRB-300005910). All participants provided voluntary, verbal informed consent prior to beginning the telephone or HIPAA-compliant Zoom interview.

## Results

### Participant characteristics

#### Stakeholders

Twenty-two stakeholders participated in an in-depth interview. Demographic data are missing from one stakeholder. The mean age was 49.4 years, and the range was 29 to 68 years. Most stakeholders were female (73%, *N* = 16), Black/African American (82%, *N* = 18), and identified as Christian Protestants (59%, *N* = 13) and heterosexual/straight (77%, *N* = 17). Most had a bachelor’s degree or higher (*N* = 19, 86%) and lived in rural areas (*N* = 13, 59%). They worked in public health, medicine, business, and community- and faith-based organizations.

#### Clients

Ten clients participated in the in-depth interviews. Demographic data are missing from one client. The mean age was 42.4 years, and the range was 26 to 77 years. All were women who identified as Black/African American. Nine (90%) identified as Christian Protestants and heterosexual/straight. Most lived in rural areas (80%) and had a bachelor’s degree or higher (70%). Eight (80%) had been previously tested for HIV at the time of the interview. Participant characteristics are summarized in Table 1.

**Table 1:** Demographic Characteristics of In-Depth Interview Participants

	**Stakeholders** ***N* = 22**	**Clients** ***N* = 10**
	**N (%)**	**N (%)**
**Age** (Mean (SD))	49.43 (10.78)	42.44 (13.81)
**Gender Identity**		
Female	16 (73%)	10 (100%)
Male	5 (23%)	0 (0%)
*Missing*	*1 (5%)*	*0 (0%)*
**Race/Ethnicity**		
Black/African American	18 (82%)	10 (100%)
White	3 (14%)	0 (0%)
*Missing*	*1 (5%)*	*0 (0%)*
**Religion**		
Protestant (e.g., Baptist, Methodist)	13 (59%)	9 (90%)
Not religious	2 (9%)	0 (0%)
Catholic	1 (5%)	0 (0%)
Prefer not to answer	4 (18%)	0 (0%)
Other	1 (5%)	0 (0%)
*Missing*	*1 (5%)*	*1 (10%)*
**Sexual Orientation**		
Heterosexual/Straight	17 (77%)	9 (90%)
Homosexual/Gay	3 (14%)	0 (0%)
Bisexual	1 (5%)	0 (0%)
*Missing*	*1 (5%)*	*1 (10%)*
**Highest Level of Education**		
High school/GED	0 (0%)	2 (20%)
Some college	2 (9%)	0 (0%)
4-year degree	9 (41%)	5 (50%)
Professional degree	8 (36%)	2 (20%)
Doctorate	2 (9%)	0 (0%)
*Missing*	*1 (5%)*	*1 (10%)*
**Residential Geographic Classification**^a^		
Rural	13 (59%)	8 (80%)
Suburban	5 (23%)	1 (10%)
Urban	3 (14%)	0 (0%)
*Missing*	*1 (5%)*	*1 (10%)*
**Employment Status**		
Full-time	17 (77%)	5 (50%)
Part-time	2 (9%)	1 (10%)
Self-employed	0 (0%)	1 (10%)
Unemployed	0 (0%)	2 (20%)
Other	2 (9%)	0 (0%)
*Missing*	*1 (5%)*	*1 (10%)*
**Occupation**		
Education/Outreach Coordinator	8 (36%)	-
CEO/Executive Director	4 (18%)	-
HIV Prevention Program Manager	4 (18%)	-
Physician	2 (9%)	-
Hospital Administrator	1 (5%)	-
Faith Leader	1 (5%)	-
Social Worker	1 (5%)	-
*Missing*	*1 (5%)*	*-*
**Ever Tested for HIV**		
Yes	-	8 (80%)
No	-	2 (20%)
*Missing*	*-*	*0 (0%)*

^a^Self-identified

**Fig. 3:**
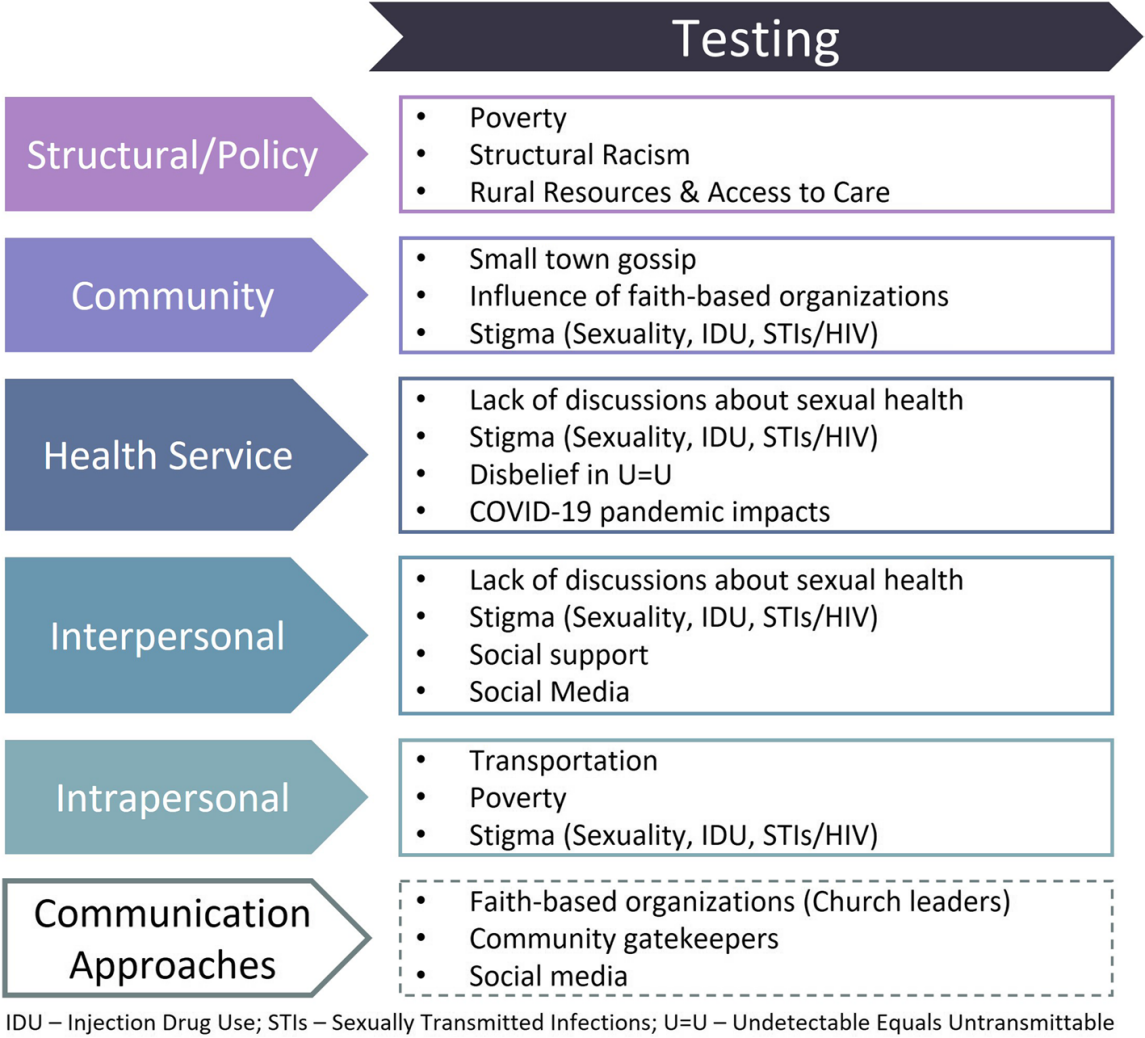
Adaptation of the Framework for Health Communication Across the HIV Care Continuum [28]

**Fig. 4:**
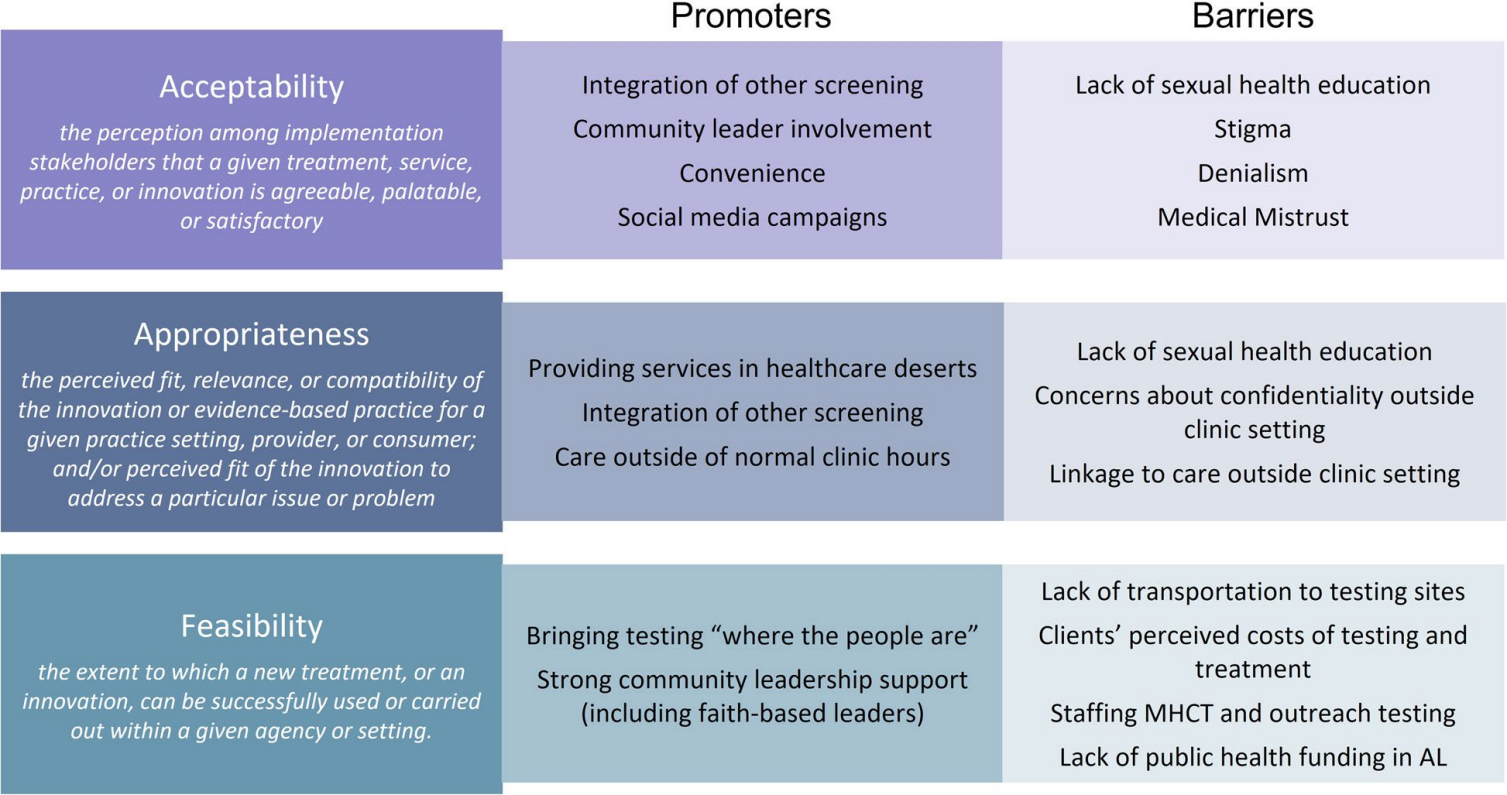
Promoters of and Barriers to Novel HIV Testing Strategies by Acceptability, Appropriateness, and Feasibility [26]

In the following sections, we describe how cultural norms, local history, racism, and poverty shape discussions around sexual health and HIV in Alabama communities. We examine how the interactions between these elements may produce barriers to HIV testing in this setting and discuss potential promoters of testing centered around community engagement. Finally, we examine the acceptability, feasibility, and appropriateness of MHCT, home testing, and the secondary distribution of self-testing kits among rural Alabama residents, both from the perspective of community stakeholders and community members. Figure 3 maps the constructs that emerged from these in-depth interviews to the HIV testing section of the Framework for Health Communication Across the HIV Care Continuum [28]. Figure 4 summarizes the promoters of and barriers to the acceptability, feasibility, and appropriateness of novel HIV testing interventions like self- and mobile-testing.

### Theme 1. Cultural norms, racism, poverty, and rurality contribute to medical mistrust and low medical literacy, impairing seeking of and access to healthcare

Economic instability and lack of health insurance lead to perceived and real barriers to HIV testing in rural Alabama. Participants describe how HIV testing is not a priority when basic needs remain unmet.

“[In the Black Belt], they’re concerned about economics more so than about testing [for HIV]. Many say, ‘If I get sick and die, I get sick and die, but I wanna be able to live. I wanna be able to have my own roof over my head. I wanna be able to have food on my table. I wanna be able to have my children to have clothes on their back. If I die, it’s all right. I die. I die, but I still wanna have some things here.’” – Stakeholder DM09 (Male, age 60)

Lack of reliable transportation options is a barrier to testing, especially in sparsely populated areas where public transportation is nonexistent and where asking for a ride from friends or family may lead to uncomfortable questions about the purpose of the clinic visit. Participants worry about being the topic of gossip in small communities, but preferences to test outside of their hometown are thwarted because of transportation challenges.

“If there was transportation, it would make it much easier, ‘cause a lot of people don’t drive. Myself, I don’t drive.” – Client AT47 (Female, age 78)

“I think if we had the capability of picking up people or going to where they are to test them, that would greatly increase testing... [Clients] don’t have the transportation to get here. If they could get transportation, they don’t want them to bring them here [AIDS service organization]... I have seen clients travel from other counties for treatment without any type of transportation and maybe take an Uber, which is hundreds of dollars, if you’re talking about 50 miles… I could only imagine how much testing we could do if we could transport people for that.” – Stakeholder AD36 (Female, age 43)

Participants describe how fear associated with a potential positive test result reduces motivations to test due to stigma, poverty, and perceived lack of access to treatment.

“I think it’s just people not wanting to know, the fear of knowing and just making that step… It’s scary. I guess it would be life changing if they tested positive, but it’s manageable. It’s treatable, but again, it’s still that stigma like you’re just the worst person in the world, which is crazy.” – Client YD85 (Female, age 30)

Alabama’s historic and current discrimination, segregation, and racism influence clients’ willingness to engage with the healthcare system. Participants describe a deep-seated and widespread medical mistrust among Black communities of the Alabama Black Belt, driven heavily by the Tuskegee Syphilis Study of the twentieth century and negative healthcare experiences. Trust in these communities is fostered through community leadership and gatekeepers, often older individuals involved in the church.

“I think structural racism factors in a lot for how we direct our testing… Up until recently I was the only Black provider for a group of people who are 80% Black. There’s some problem with that, and that goes back into people going to medical school. It goes back to even further, that Black people are not geared, are not trained, are not exposed, are not pushed into going to medical school, so the number of Black physicians and Black nurse practitioners is limited.

There are all kinds of things that go into structural racism. The transportation issue, if you’re rich and white, you have transportation, so it doesn’t bother you, and so you don’t need to worry about the young Black person who’s trying to get to work with no car. You don’t have to worry about it, ’cause ‘I got mine.’ I think it impacts a whole lot… A lot of times, when you’re coming for testing, if you have a job that says, ‘I’m not going to let you off to go get tested,’ those tend to be the lower paying jobs, which tend to be more people of color … One of the things that I felt like I was called to do in this position was to try to talk to somebody on a level that they would understand. I’m not sure everybody does that, and so I was somewhere this weekend listening to people talk about how physicians talk down to them, and they have to come home and call me to say, ‘What did he mean by this?’ When you look at the Black Belt, people who tend be in the poor rural areas tend to be people of color, and that is very, very difficult to get them to understand why they need to be tested, and how to protect themselves, and it doesn’t mean that you’re the scum of the earth because you have either been exposed or gotten HIV. All of those educational pieces are fed by structural racism, in my opinion.” – Stakeholder EB93 (Female, age 64)

“African American populations tend to still have that medical mistrust because of systems that have not been beneficial to them. Black people have been used as experiments. They’ve been misdiagnosed. They have been forgotten about in the medical system. Yes, we have the access and the tools to get into rural counties where Black people reside and provide them the resources, but just because of how they’ve been treated throughout the years, they tend to not trust the system.” – Stakeholder DT25 (Female, unknown age)

Clients acknowledged that local history, stigmas perpetuated by cultural norms, poverty, and rurality are potential barriers to acceptability of HIV mobile- and self-testing.

### Theme 2. Lack of sex education contributes to low knowledge of HIV in communities, low risk perceptions, STI incidence, and reinforces sexuality and STIs/HIV stigmas

Deep-seated stigma towards sexuality, especially among LGBTQ + populations in southern rural Christian communities drive lack of understanding about sexual health, STIs, and HIV and stymie discussions from happening in communities, leaving residents underinformed about healthcare needs and decisions. This exacerbates limitations to care access already thwarted by barriers of transport, stigma, and racism, and could be potential obstacles to the acceptability of interventions that target sexual health.

“Even with trying to talk to parents about relating information and awareness on sexual health topics with their children, I just think that they don’t wanna talk about it. The faith community doesn’t wanna be a part of it. It’s like they think that you can only get HIV from having sex with the wrong person or whatever, and then they don’t wanna talk about sex... People will want you to come and talk about HIV, but oh my goodness, don’t use the word sex. I think that is a part of stigma.” – Stakeholder NT38 (Female, age 53)

“Well, as a Black female in Alabama, a Christian community, I guess [accessing sexual health services] is looked down upon to a certain extent. ‘Cause the mentality is that, if you’re married and you’re having sex, then certain things shouldn’t happen…” – Client EP37 (Female, age 44)

Without comprehensive, nonjudgmental discussions about sexual health and HIV prevention, stigma continues to dissuade individuals from accessing testing, both because they perceive it as unnecessary for themselves and because they fear judgment from their communities and peers.

“Just talk to people in the community to make it more of a conversation, as opposed to, ‘This is something that has to be done,’ because people have to feel like they can trust people to have conversations like this. It’s more of a trust thing and a discretion thing. If you can create both of those, people be more likely to get tested.” – Client DM14 (Female, age 34)

“Some people don’t feel like they need to get checked. They feel like they can trust the person who they’re having sex with. I feel like, if you're having sex, you should get tested 'cause everyone is at risk. You have some people who get it done freely, but some don’t—they don't like to do it.” – Client YD85 (Female, age 30)

Healthcare providers are not impervious to the intersecting stigmas. Stakeholders describe their frustrations as they have advocated for regular opt-out testing in emergency rooms, primary care clinics, and other routine-care practices in their communities. Providers in rural areas have often known their patients and families for many years, leading to discomfort discussing sexual activity at all, especially topics like HIV prevention.

"Physicians, health care providers, have got to educate themselves on PrEP, on HIV, on all cultural competencies, things like that. That way, people will be more comfortable discussing these things. You don’t want to discuss things with a doctor, and they’re going to stigmatize you because you gave them your honest truth... A lot of health care providers don’t even realize the number of HIV cases in their area. You’ve got to know this information. Small towns, you go to a doctor. It may be your doctor since you were a baby. Now you’re 21. The doctor doesn’t discuss with them sexual health or risk because they feel like that’s not appropriate... As a doctor, it’s got to be your responsibility to make sure that you’re seeing this whole person… We’ve got to get away from that and start educating and opening up." – Stakeholder AD36 (Female, age 43)

“I think maybe the most important thing would be to get people to realize that HIV testing is just as essential as being tested for diabetes, or hypertension. If we could get to that state, then I think that would make it easier. … People are still very ignorant of how HIV is transmitted, and things of that nature. Because the provider for the most part, most of the providers don’t just ordinarily offer the HIV testing, even though we talk with ‘em, and push the issue, a lot them - making it just a regular part of care - it just doesn’t happen.” – Stakeholder LM46 (Female, age 55)

### Theme 3. Messaging about “Undetectable = Untransmissible” (U = U) is not well understood or believed in communities

Many stakeholders and clients discuss how U = U may be helpful to ameliorate internalized stigma among people living with HIV, but it does little to dissuade community stigma.

“People do not understand [U=U]. A lot of people who have HIV don’t even understand it. I don’t personally think U=U helps stigma. I think it helps the person who has HIV and maybe their support system. I think it helps if you are told U=U, and you’re in a relationship with somebody [living with HIV]… When I teach this to people all the time, they tell me, ‘Oh, well, I don’t believe that. I wouldn’t trust it.’” – Stakeholder AD36 (Female, age 43)

“In the men that have sex with men community, that message [U=U] is received loud and clear. There is still that fear. They get it, they understand it. They talk to their friends about it. The ones that are living with HIV talk to their partners about U=U. There’s still that fear associated with transmitting HIV, what comes after that. I think it’s only really talked about in certain pockets of communities. If I was to approach a person that was in the young Hispanic community, they probably wouldn’t even have heard the term U=U. It’s very rare that I come across a heterosexual black person that’s heard the term U=U. I think it’s really only in certain pockets where that message is talked about often.” – Stakeholder DT25 (Female, unknown age)

Clients discuss how the message, even when understood, is not enough to overcome the stigma.

“I follow some people on social media that are undetectable, and they talk about stigma, and how people don’t understand what that means that they can’t transmit it [HIV], and how it’s hard to date, and how they feel like you shouldn’t just set yourself up for one group of people to date. You should be able to date anybody, and they should be able to understand hey, this is undetectable. This is what we can do for you not to contract it and stuff like that. I think that’s hard. [crying] That’s hard. That’s hard. Even though it’s undetectable, it’s still that stigma. That scarlet letter—I don’t know what it is with HIV. I just think it’s going to always have that stigma.” – Client YD85 (Female, age 30)

Others agree that the internalized stigma felt by PLWH may be lessened by knowing their viral load is undetectable but still have concern that U = U is not enough to protect the partners of PLWH from acquiring the virus.

“[U=U] may make some people feel comfortable after being diagnosed, may make them feel like a person again… I don’t feel like it’s okay [to stop prevention measures] because it’s undetectable. I really don’t because if you have a partner, y'all are going to get tested, and then they're taking the pill, and it comes back undetectable, but at the same time, if your partner stops taking their medication, then, hey, you're at risk. You know what I mean? You're at risk all the time, but still, you have more faith that they tested negative. It can be a lot as far as that situation.” – Client JN94 (Female, age 32)

Ultimately, both stakeholders and clients agree that U = U may not be the most powerful tool in stigma reduction. They suggest other messaging, in addition to U = U campaigns.

“[The message should be] that everybody should be tested regardless. Regardless of your history, your risk factors, everybody regardless of your race, your ethnicity, regardless of your sexual orientation. I think if we made it a norm that everybody’s tested, I think that would help.” – Stakeholder AD36 (Female, age 43)

“If you test negative, you can take a pill and prevent HIV. If you test positive, most people are on one pill a day with normal, healthy lives and are not transmitting HIV. Those are game changers in people’s understanding and willingness to engage with HIV testing or treatment, I think.” – Stakeholder ED49 (Female, age 63)

### Theme 4. Community involvement, particularly among faith leaders, is needed to promote communication and trust between communities and testing advocates

Stakeholders and clients describe how some members of their communities that drive HIV education and prevention overcome barriers to testing to empower individuals to get tested and spread accurate information.

“We have what we call community gatekeepers, which are people in the community who we work with and who have a good rapport and respect with the community that we serve and that they live in, so when we do have the opportunity to have events or offer testing that we make sure they go out, and they speak with people in the community and let them know ahead of time and also have just regular conversations about HIV with them, just say, ‘Hey, you don’t know—you won’t know your status until you get tested.’ They can have those conversations that we can’t.” – Stakeholder YC13 (Male, age 42)

“I think it’s so important to the grassroot. Advocates really meeting constituents where they are. If you meet them where they are at every level, you now are connecting to them where they are: you will connect to the church, you will connect to the political leaders, you will connect to the family members, you will connect to the people there 'cause you're there seeing them get help and want help.” – Stakeholder OM68 (Male, age 50)

“Just make sure you’re reaching out to the right people to get people to come out to do things and just creating trust first and discretion. If you could get some of those mobile units on the actual ground and, like I said, get the right community leaders out—they could use the mayor and other people to get other people to come out, but you have to start with finding those people that are respected in the community that actually work in the community to get folks out.” – Client DM14 (Female, age 34)

Many of these “gatekeepers” are church leaders who advocate for health education in their faith-based organizations. Stakeholders describe faith-based organizations as hubs of civil rights and social justice movements, which are capable of reaching many people through weekly gatherings.

“Historically in Alabama, Black people follow what their minister says. Historically, the places where civil rights leaders met, gathered, and mobilized people were the churches. There are still a lot of Black people who will listen to what their minister says, and so I think if we can get the ministers on board... That’s kind of where the disconnect is. I think if we can continue to educate them, and they will talk to their parishioners, then we can get testing done. Put something in the bulletin. There could be educational pieces in the lobby. There could be participation when the churches have little fairs, little outdoor activities… So, I think the churches can play a big role. It would take not only the minister, but it would take some deacon, deaconess, those key people in the church community to be on board, to be educated themselves and to push the educational piece.” – Stakeholder EB93 (Female, age 64)

“We can reach so many people if we can get the faith-based community on board. Also, faith-based communities have influenced a lot of the thinking around HIV and sex and all of these negative connotations sometimes that it has. If we can get faith-based leaders that are on board to start changing the narrative, that’s another way that we can start chipping at that stigma too.” - Stakeholder NH76 (Female, age 32)

Other stakeholders and clients discuss the important role of social media in spreading information about HIV and sexual health, and the importance of having influencers on social media discussing HIV.

“Social media is golden. It could work wonders for HIV messaging, getting information out about testing. You have to have an influencer. You can’t just have a regular [person]—I can’t just tweet about it. I don't have that type of following. You have to have someone who has the following who can get the attention of these people. Like I said, an influencer. That's what they're called. That's their job.” – Stakeholder NH76 (Female, age 32)

“Everybody’s on social media, but finding a good, positive, and informative way to post it [educational and testing information] on social media, that makes people comfortable.” – Client AJ60 (Female, age 27)

Participants also describe how social media is also an effective tool for disseminating information about outreach testing events like mobile testing.

### Theme 5. Novel testing strategies such as home-, self-, and mobile-testing are acceptable to many clients and stakeholders and may encourage testing by diminishing barriers like transportation, confidentiality, and convenience

Most clients and stakeholders showed enthusiastic support for mobile-based HIV counseling and testing, describing it as “awesome” (SAD36), “accessible” (SLT95), and “convenient” (SLT95, SYK48).

“If we had a mobile testing unit, oh my gosh, the places we could go and the things we could do! If I had unlimited funding, that would be one of the main things that we would do, is fund a testing unit.” – Stakeholder AD36 (Female, age 43)

“I think [mobile testing] is an excellent idea ‘cause we have a lot of patients that are homeless, no transportation. Our biggest thing here in the clinic is our patients getting here. If we can make ourselves accessible to getting to them, that would be good. That’d put a lot of that no testing down. I’m excited about that. I can’t wait.” – Stakeholder AM83 (Female, age 52)

“I really think you may get more numbers, get more people out tested if you do a mobile. It’s an easy access to get people tested by them not actually having to make an appointment or something like that. If they see the bus, they can just stop by while they're out.” – Client JN94 (Female, age 32)

Participants highlighted the importance of confidentiality.

“If it's set up in high traffic areas, I don't think a lot of people would participate in it, from the fear of people knowing or seeing them go in there or have it done, but I think it'll be beneficial in the low-traffic areas or a set up at a place away from it where people won’t be in fear of being identified or people speculating certain things about…” - Client AM60 (Female, age 41)

“We’re going to pull up in the mobile vehicle and we got to have something on there to advertise who we are. People are going to be like, ‘Oh gosh, here we go again. I’m going to be seen getting into the mobile testing unit.’ I do think that will be a barrier, but I think it’s one we could overcome.” – Stakeholder AD36 (Female, age 43)

Clients again mentioned how important community “gatekeepers” are in the promotion of testing.

“I feel like certain people may not show up for things like that. You have to get a trusted community member to get people to come out for something like that. I think it’s a great idea. You just going to have to get somebody that the community respects to get actual people to pull up. You have people that are just fearful of having people in their business...” – Client DM14 (Female, age 34)

Most suggested advertisings for and offering multiple health screenings, like those for COVID-19, diabetes, and cancer, to improve acceptability, appropriateness, and feasibility of HIV testing in their communities. Offering additional health screenings provides an incentive for clients to access HIV testing by making it more convenient and less stigmatized.

“I would love to offer more than HIV testing because that gets people to get tested. Most people don’t just want to come get tested for HIV. They want gonorrhea, chlamydia, syphilis, all that good stuff. Give me the ability to test for more things. I think that would be great.” – Stakeholder AD36 (Female, age 43)

“Maybe if they can do some regular testing, say, “Hey, we’re doing a health screening,” and AIDS was just part of it, so nobody knew if you got it or not, then that would be a way where people could get tested that really care about people just knowing this mobile is just for AIDS. If you’re just saying, “Hey, this is a mobile for a health screening. Come on in,” and when they get in there, you can privately ask them, “A part of our screening is being screened for HIV,” then they would probably more likely take that test, because it’s not just a mobile for to get screened for that.” – Client EM00 (Female, age 49)

“I’ve always believed [HIV testing] needs to be part of a general screening, and I think we need to tout that to not just the community but to the medical community, and so there would be more testing in doctors’ offices. I was a private physician for close to 25 years, and I know how hard it is. I understand the reluctancy to live in the community and test your lady that you go to church with. I got it, but the fact of the matter is we have to, and the more we talk about it, and the more we normalize it for both clients as well as the medical profession, the more it will be done and the less we’ll have undiagnosed HIV patients running around.” – Stakeholder EB93 (Female, age 64)

Additionally, many participants were agreeable to the distribution of self-testing kits within their communities but highlighted the importance of guidance on how to conduct the testing as well as pre- and post-testing support and linkage to prevention and care. Stakeholders were concerned about patients testing positive and “running” (SNH76), or not contacting the clinic to link to care.

“The pro is the privacy. I can do this, and I never have to walk into a building. I can just go buy it, or I can get it mailed to me. I do this in the privacy of my home. No one knows. Con, just like the person that waited for months to come in, there’s no one there to guide you when this comes back negative or positive… Negative, you still haven’t got any education on preventing. Positive, there’s no one there. How do you accept this? We don’t know what’s to keep somebody from harming themselves because they’re not educated enough on it to know that you’re going to live a long, healthy life once you get into care. That’s probably one of my biggest fears, is that someone will hurt themselves because they feel their life is over because they’re there alone at that moment. That’s the con to me and the lack of education. You can put something in a pamphlet all day long, but that doesn’t make somebody read it.” - Stakeholder AD36 (Female, age 43)

“I think [self-testing] is good. Then you don’t really have to be concerned about, ‘Oh, they saw me here, or I gotta go there,’ or watching your back like, ‘Is somebody going to see me?’ I think that’s awesome. Privacy, in your own home, do it yourself. You don’t have to have anyone else involved… Possibly [a barrier would be] people not doing it properly, someone doing something wrong and not getting it right, that type of thing, but that would probably be the only thing. That’s positive especially if you’re dealing with people who otherwise would not go to be tested on their own or who would not feel comfortable doing that.” – Client EP37 (Female, age 44)

Overall, both clients and stakeholders were amenable to new HIV testing interventions and thought their communities would be accepting of more options, as long as opportunities for more discussions and accurate information about HIV were regularly presented.

## Discussion

In this study, we sought to explore barriers to and promoters of the first EHE pillar, HIV testing, in the rural Black Belt region of Alabama from the diverse perspectives of stakeholders and community members. We found that intricately linked effects of cultural norms, racism, rurality, and poverty have led to a lack of awareness of HIV, limited sexual health discussion, and ultimately contribute to poor healthcare seeking behaviors and low HIV testing uptake within these communities. Stigma related to sexuality, STIs/HIV, injection drug use, and sexual activity, often driven by religious norms, create barriers to testing as clients navigate confidentiality and small-town gossip. Stakeholders and clients emphasized the importance of finding community “gatekeepers” to promote sexual education and HIV testing within small rural communities, and most suggested faith-based leaders as the primary entrance into communities, as changing the dialogue related to sex in the church may increase acceptability and feasibility of normalized HIV testing. When clients and stakeholders were asked about testing strategies novel to rural Alabama, such as mobile- and self-HIV-testing, most were supportive, deeming the strategies as appropriate to address several of the challenges associated with poor HIV testing in the Black Belt and other rural regions.

While our study is unique in its focus on exploring barriers to and promoters of HIV testing within the Black Belt region, our findings echo some of the barriers to rural HIV testing documented in previous studies. A study of HIV testing and treatment services in rural counties of 10 southern states found transportation to be a significant barrier, with the average distance to treatment at 50 miles [19]. Additionally, facilitators of testing included integrating HIV testing with other services and making testing easily accessible [19]. Other studies of the rural south find lack of risk perception prevents clients from accepting testing [20, 21, 31], but routine implementation of opt-out testing may mitigate some of these barriers [21]. Other studies examined the effects of intersecting stigma on HIV testing. Arnold et al. found homophobia and HIV-related stigma were intertwined with churches and families within participants’ Black communities, leading to reluctance to engage with HIV testing [32]. A New York City study examined the intersecting racism and classism experienced by Black and Latinx PLWH who had disengaged from care and explained public health’s potential over-emphasis on the Tuskegee Syphilis Study, which may discount current ongoing social inequities and lived experiences [33]. While this study explores participants’ experiences in an urban metropolis, our findings may indicate additional layers of stigma and racism, as southern rural communities, inclusive of those surrounding Tuskegee, Alabama, have a deep history of slavery, segregation, mistreatment, and continued structural racism that influence medical mistrust, misinformation, and HIV-related beliefs.

Our findings provide a framework for implementation of mobile-based HIV counseling and testing in rural Alabama. Participants described their preferred sites for mobile testing, including parks and churches, and suggested mobile testing could increase HIV testing uptake. Importantly, participants also expressed the need for mobile vans to provide other non-HIV-related services and to integrate and link to primary care. This finding is consistent with a recent qualitative study by Henkhaus et al., where key stakeholders recruited from an infectious disease clinic and surrounding agencies in Atlanta, Georgia, highlighted the need to integrate mobile health clinics with other care delivery such as telemedicine [34]. Participants did not feel that integration of U = U messaging would promote uptake of testing due to low knowledge and/or mistrust of U = U tenets. Other studies observe misunderstanding and perceived lack of acceptance of U = U among the general population [35–37], but many find that U = U may be beneficial in reducing stigma in some communities [38]. As some stakeholders noted, current campaigns and messaging around HIV have been limited to specific key populations, like men who have sex with men, but could be broadened to reach more groups, especially older generations. Better messaging and communication around U = U may help to facilitate discussions around HIV in the community, but currently does little to improve community stigma. Additional messages may work better to reduce stigma and should include discussion around universal and opt-out testing, as well as breaking down U = U into discussions about medication management and PrEP for prevention.

Some strengths of this study include rich perspectives from stakeholders and clients living within the Black Belt and other rural areas in Alabama. We employed a rapid qualitative analysis approach to provide timely and valid findings to influence the implementation stage of a mobile-based HIV testing program and contribute to the growing literature of this analysis technique. To reduce the possibility of bias, we undertook a number of steps during data collection and analysis including peer debriefing and triangulation. We also used the CFIR framework to guide analysis. However, like other qualitative studies, our study findings may not be as generalizable to places outside the rural Deep South, including urban and suburban areas. Our study was also limited by the small sample size, especially our client sample which consisted of only Black straight women. While this is an important, uniquely vulnerable population to HIV infection and vital members of their communities, this limits the generalizability of our data. Possible reasons for the sample consisting of this population include recruitment accessibility, community networks, and willingness to discuss sexual health and HIV. We recruited clients mainly from a rural health fair, which occurred midday, and the population attending was primarily Black women. Additionally, stigma related to HIV may be more prevalent among Black men, which was discussed by many clients and stakeholders.

## Conclusions

Diverse perspectives from stakeholders and community members in rural Alabama show many barriers to HIV testing, including stigma, poverty, racism, low risk perception, as well as opportunities for promoting testing through integration with other health screenings, universal opt-out testing, improved community discussions about sex, and implementation of testing strategies novel to the region, like mobile- and self-testing. The analysis of these in-depth interviews informs the implementation of a mobile testing van within 28 rural counties in Alabama, with an emphasis on community involvement, feedback, and discussion. The use of novel HIV testing strategies must be continuously evaluated, monitored, and tailored to the needs of specific populations and communities, taking into consideration the historic and current climate of structural racism, classism, and medical mistrust. Engagement with community “gatekeepers,” especially faith-based leaders, is crucial to building trust within communities, which can increase the uptake of HIV testing through increased discussion, knowledge, and empowerment.

## Supplementary Information

**Additional file 1.** Interview Guide for Key Stakeholders.

**Additional file 2.** Interview Guide for HIV Testing Clients.

**Additional file 3.** Big Data Stakeholder Interview Summary.

**Additional file 4.** Big Data Client Interview Summary.

## Data Availability

The dataset supporting the conclusions of this article is not available as participants did not provide consent for transcripts to be publicly available.

## References

[1] CDC (2018). HIV Surveillance Report, 2017.

[2] CDC. HIV in the United States by Region 2018 [Available from: https://www.cdc.gov/hiv/statistics/overview/geographicdistribution.html.

[3] Scarinci IC, Moore A, Benjamin R, Vickers S, Shikany J, Fouad M (2017). A participatory evaluation framework in the establishment and implementation of transdisciplinary collaborative centers for health disparities research. Eval Program Plann.

[4] Zekeri AA (2019). Food insecurity and maternal mental health among African American single mothers living with HIV/AIDS in the Alabama black belt. J Health Care Poor Underserved.

[5] Zekeri AA (2013). Educational attainment and self-rated health status among single mothers in rural Alabama. Psychol Rep.

[6] Zekeri AA, Habtemariam T, Tameru B, Ngawa D, Robnett V (2009). Conspiracy beliefs about HIV/AIDS among HIV-positive African-American patients in rural Alabama. Psychol Rep.

[7] Johnson ER, Carson TL, Affuso O, Hardy CM, Baskin ML (2014). Relationship between social support and body mass index among overweight and obese African American women in the rural deep South, 2011–2013. Prev Chronic Dis.

[8] University of Alabama Center for Economic Development. Alabama’s Black Belt Counties [Available from: http://www.uaced.ua.edu/uploads/1/9/0/4/19045691/about_the_black_belt.pdf.

[9] Alabama Department of Public Health, Office of HIV Prevention and Care, HIV Surveillance Branch (2019). 2019 State of Alabama HIV Surveillance Annual Report.

[10] Alabama Department of Public Health, Office of HIV Prevention and Care, HIV Surveillance Branch (2018). State of Alabama HIV Surveillance 2018 Annual Report.

[11] U.S. Department of Health and Human Services, Office of Infectious Disease and HIV/AIDS Policy. What is Ending the HIV Epidemic in the U.S.? 2021 [Available from: https://www.hiv.gov/federal-response/ending-the-hiv-epidemic/overview.

[12] Alabama Department of Public Health, STD and HIV Surveillance Branch (2019). HIV/STD Integrated Epidemiological Profile, 2018.

[13] CDC (2019). HIV in the Southern United States. Issue brief.

[14] Pitasi MA, Delaney KP, Brooks JT, DiNenno EA, Johnson SD, Prejean J (2019). HIV Testing in 50 Local Jurisdictions Accounting for the Majority of New HIV Diagnoses and Seven States with Disproportionate Occurrence of HIV in Rural Areas, 2016–2017. CDC MMWR Morb Mortal Wkly Rep.

[15] Matthews L, Long D, Nassell A, Heath S, Jackson E, Gordon B, et al. Using big data to estimate to inform testing interventions in Alabama. National Ending the HIV Epidemic Meeting, Scientific Session 3, DIAGNOSE & RESPOND Panel; 2021. https://isc3iold.isgmh.northwestern.edu/2021-national-ehe-meeting/.

[16] Dailey AF, Hoots BE, Hall HI, Song R, Hayes D, Fulton P (2017). Vital signs: human immunodeficiency virus testing and diagnosis delays - United States. MMWR Morb Mortal Wkly Rep.

[17] Satcher Johnson A, Song R, Hall HI (2017). Estimated HIV incidence, prevalence, and undiagnosed infections in US States and Washington, DC, 2010–2014. J Acquir Immune Defic Syndr.

[18] Hood KB, Hall CJ, Owens BD, Patev AJ, Belgrave FZ (2020). HIV testing behaviors among black rural women: the moderating role of conspiracy beliefs and partner status disclosure. Ethn Dis.

[19] Sutton M, Anthony MN, Vila C, McLellan-Lemal E, Weidle PJ (2010). HIV testing and HIV/AIDS treatment services in rural counties in 10 southern states: service provider perspectives. J Rural Health.

[20] Ratcliff TM, Zlotnick C, Cu-Uvin S, Payne N, Sly K, Flanigan T (2012). Acceptance of HIV antibody testing among women in domestic violence shelters. J HIV AIDS Soc Serv.

[21] Wise JM, Ott C, Azuero A, Lanzi RG, Davies S, Gardner A (2019). Barriers to HIV testing: patient and provider perspectives in the deep south. AIDS Behav.

[22] Habtemariam T, Tameru B, Nganwa D, Beyene G, Ayanwale L, Robnett V (2008). A conceptual multivariate modelling approach for integrating epidemiologic and psychosocial determinants to examine the epidemiology of diseases in under-served populations. Adv Syst Sci Appl.

[23] Kempf MC, Ott C, Wise JM, Footman AP, Araya BY, Hardy CM (2018). Universal screening for HIV and Hepatitis C Infection: a community-based pilot project. Am J Prev Med.

[24] Rice WS, Stringer KL, Sohail M, Crockett KB, Atkins GC, Kudroff K (2019). Accessing pre-exposure prophylaxis (prep): perceptions of current and potential PrEP users in Birmingham. Alabama AIDS Behav.

[25] Aholou TM, Cooks E, Murray A, Sutton MY, Gaul Z, Gaskins S (2016). "Wake Up! HIV is at Your Door": African American faith leaders in the Rural South and HIV perceptions: a qualitative analysis. J Relig Health.

[26] Weiner BJ, Lewis CC, Stanick C, Powell BJ, Dorsey CN, Clary AS (2017). Psychometric assessment of three newly developed implementation outcome measures. Implement Sci.

[27] United States Census Bureau. Geography program: glossary: urban and rural 2019 [Available from: https://www.census.gov/programs-surveys/geography/about/glossary.html#par_textimage_29.

[28] Babalola S, Van Lith LM, Mallalieu EC, Packman ZR, Myers E, Ahanda KS (2017). A Framework for Health Communication Across the HIV Treatment Continuum. J Acquir Immune Defic Syndr (1999).

[29] Hamilton AB, editor. Qualitative methods in rapid turn-around health services research veterans affairs health services research & development cyberseminar spotlight on Women’s Health. 2013.

[30] Damschroder LJ, Aron DC, Keith RE, Kirsh SR, Alexander JA, Lowery JC (2009). Fostering implementation of health services research findings into practice: a consolidated framework for advancing implementation science. Implement Sci.

[31] Harmon JL, Collins-Ogle M, Bartlett JA, Thompson J, Barroso J (2014). Integrating routine HIV screening into a primary care setting in rural North Carolina. J Assoc Nurses AIDS Care.

[32] Arnold EA, Rebchook GM, Kegeles SM (2014). 'Triply cursed': racism, homophobia and HIV-related stigma are barriers to regular HIV testing, treatment adherence and disclosure among young Black gay men. Cult Health Sex.

[33] Jaiswal J, Singer SN, Siegel K, Lekas HM (2019). HIV-related 'conspiracy beliefs’: lived experiences of racism and socio-economic exclusion among people living with HIV in New York City. Cult Health Sex.

[34] Henkhaus ME, Hussen SA, Brown DN, Del Rio C, Fletcher MR, Jones MD (2021). Barriers and facilitators to use of a mobile HIV care model to re-engage and retain out-of-care people living with HIV in Atlanta, Georgia. PLoS ONE.

[35] Rendina HJ, Cienfuegos-Szalay J, Talan A, Jones SS, Jimenez RH (2020). Growing acceptability of undetectable = untransmittable but widespread misunderstanding of transmission risk: findings from a very large sample of sexual minority men in the United States. J Acquir Immune Defic Syndr.

[36] Rendina HJ, Talan AJ, Cienfuegos-Szalay J, Carter JA, Shalhav O (2020). Treatment is more than prevention: perceived personal and social benefits of undetectable = untransmittable messaging among sexual minority men living with HIV. AIDS Patient Care STDS.

[37] Rivera AV, Carrillo SA, Braunstein SL (2021). Prevalence of U = U awareness and its association with anticipated hiv stigma among low-income heterosexually active black and Latino adults in New York City, 2019. AIDS Patient Care STDS.

[38] Bor J, Fischer C, Modi M, Richman B, Kinker C, King R (2021). Changing knowledge and attitudes towards hiv treatment-as-prevention and "Undetectable = Untransmittable": a systematic review. AIDS Behav.

