# Supplementary material for: Rapid qualitative analysis approach to stakeholder and client interviews to inform mobile-based HIV testing in the U.S. Deep South
Source: Arch Public Health. 2023 Feb 15;81:24. doi: 10.1186/s13690-023-01039-w (PMC9930722; doi:10.1186/s13690-023-01039-w)
Supplement: Supplementary file 1 — Additional file 1. Interview Guide for Key Stakeholders. [file 13690_2023_1039_MOESM1_ESM.docx]

Big Data Study

Interview Guide for Key Stakeholders

| PARTICIPANT ID: | DATE (MM/DD/YY): | START TIME: | END TIME: | INTERVIEWER: |
| --- | --- | --- | --- | --- |

Reminder to interviewers:

Questions in the left-hand column will be asked of all participants, while questions in the right-hand columns are probes, and are to be asked only to help the participant describe their experience or if the participant does not bring up these topics spontaneously.

Interview script:

Hello, my name is [INTERVIEWER NAME]. I am from the University of Alabama at Birmingham (UAB). We are conducting interviews with healthcare providers and key stakeholders around Alabama to better understand the barriers and facilitators to HIV testing in Alabama.

As a reminder, you are not required to answer my questions, and you may skip any questions that make you uncomfortable. If you decide that you no longer want to participate in this interview, we can end at any time. As discussed, I will use a digital recorder to record our conversation.

What questions do you have before we begin the interview?

(TURN ON DIGITAL RECORDER)

| *Topics and Main Questions* | *Probes* |
| --- | --- |
| **General HIV Information** | |
| I am going to ask you questions relating to HIV care in the places where you work and live. There are no right answers—we are looking to you as the expert for your thoughts and opinions. | |
| Please tell me a little about any of your roles providing or supporting HIV testing and/or HIV care and prevention services within your community. |  |
| What are the places where clients can access HIV testing near you? Can you tell me a little about the pros and cons of the different sites and options? |  |
| What are some factors that make it easier for people to access HIV testing in your setting? |  |
| **What are some factors or barriers that limit provision of routine HIV screening and testing in your practice and community?** | Probe the following:  *Provider factors:* time constraints, competing priorities, proper counseling spaces, language barriers  *Client factors:* Cost concerns, stigma, racism, Structural barriers (transportation, insurance, etc.), Legal barriers, like the illegality of sex work and injection drug use, Low risk perceptions, Health literacy, Poverty, Lack of trust in healthcare system |
| How do you think cost concerns impact whether clients access testing? | **Are clients offered incentives to complete HIV testing?**  How is HIV testing funded at your location/testing sites you are familiar with? Do clients have to pay a price or copay to be tested? |
| What is the routine if a client tests positive for HIV? | What is the procedure for getting them started on treatment? |
| What is the routine if a client tests negative for HIV? | What are the opportunities for HIV risk counseling or access to PrEP care?  What additional resources can they be linked to? |
| Who tends to access testing for HIV where you work / testing sites you are familiar with?^1^ | Can you tell me a little about any groups who might benefit from testing but do not access services?  Do most of the people who get tested at your location live near there? What setting is the location (urban, rural, suburban)? |
| How have testing practices changed since you started working at this location?^1^ | What kinds of changes have occurred?  Why did these changes happen?  How do you think the changes have impacted access to HIV testing, counseling, and care?  How does the leadership at your organization respond to change? |
| What are some ways that the COVID-19 epidemic could affect or is affecting HIV testing? | Positive impacts?  Negative impacts? |
| How might misinformation impact whether and how people access HIV testing? | How do you dispel/address myths and misconceptions about HIV testing with your clients? |
| How do you think race impacts HIV testing in Alabama? | Can you think of things providers, healthcare systems, community leaders could do to improve adverse impacts of race on HIV testing or increase the rate of HIV testing in Black/African American communities? |
| How do you think the history of medical malpractice and medical mistrust impacts HIV testing among black or African American populations? | Can you think of things providers, healthcare systems, community leaders could do to improve adverse impacts of healthcare mistrust on HIV testing?  Probe:   - Stigma - Racial/ethnic HIV disparities - Structural barriers to prevention - Social injustice   What role should communities play in reaching vulnerable populations? |
| **Maps Feedback**  *Interviewer to present the participant with an electronic version of most updated map of HIV testing incidence by ZIP Code and HIV prevalence by county.* | |
| Here is a map showing the prevalence of HIV in Alabama by county as well as testing incidence by the zip code level. | |
| What are your thoughts about the patterns we are seeing? | Do you have thoughts about why we see these patterns?  Why do think testing isn’t happening in these areas?  Why do you think testing is happening in these areas? |
| Where do you think we should be focusing outreach efforts to improve efforts? | Tell me about areas on this map where you think it might be easier to improve testing.  Tell me about areas on this map where you think it might be more challenging to improve testing. |
| **Perceived Barriers and Promoters** | |
| **(This question is for clinical providers who we interview)** What factors affect your ability to provide HIV testing, as a provider/organization? | How do these factors affect your ability to provide testing? |
| **(This question is for clinical providers who we interview)** Can you think of anything that would make it easier for you to provide HIV testing as a provider/organization? | How would these things make it easier? |
| How could community members help inform policy or support providers to increase HIV testing in rural Alabama? |  |
| How has your organization worked to include community perspectives? | What has worked? What has been more challenging? |
| Which community leaders might be able to provide more information to our research team about strategies to improve HIV testing in your setting? | **How are faith-based organizations involved with HIV testing in your community?** |
| How do you think messages about how people living with HIV can live a long life on modern treatments might impact stigma? | How do you think this message is being heard or understood in the community? |
| How do you think messages about how undetectable is untranmissiable (U=U) could impact stigma? | How do you think these messages are understood in the community?  What might be some ways to better share these messages with people living with or at risk for acquiring HIV? |
| How do you think the availability of effective HIV prevention (e.g. PrEP) might encourage people with HIV risk to access testing and preventative care? |  |
| Can you think of other messages that might help people in rural Alabama overcome barriers to testing? |  |
| Leaders at the White House, the CDC, Department of Health and Human Services and the NIH have come together to push to end the HIV epidemic in the US. Data suggest that only about 30% of adults living in rural counties in Alabama have ever tested for HIV. How do you think this funding could be used to improve HIV testing uptake in rural Alabama? | Why would these things make it easier?  **What role does the community play in educating around HIV prevention?**  **What role does it play in building trust and buy-in (people understanding the importance of testing for HIV)? (coalition building, collaborative networks, advisory groups, and partnerships, etc.)** |
| What role does social media have on the uptake of HIV testing? | **What are other marketing and awareness ideas to engage high risk and rural communities to get tested for HIV?** |
| **MOBILISE – Mobile Testing Questions**  We are working with the Medical Advocacy and Outreach (MAO) in Montgomery to evaluate their implementation of a mobile-testing unit. The next questions will inform this implementation of mobile-based HIV testing in rural Alabama. | |
| Tell me about your experiences with mobile-based HIV counselling and testing (MHCT) in Alabama.  *If the participant has not had any experiences with mobile-based care, move on to next question.* | What made MHCT easier, more efficient, or a better experience?  What were some challenges that you experienced with MHCT?  Can you describe the types of sites where the MHCT was held?   - What sites worked well? Why? - What sites didn’t work very well? Why? - What types of site are good for reaching communities most effectively? - Which sites have the highest number of people getting tested? - Which sites are impacted by community stigma? Which sites are less impacted by stigma? - Which sites are most comfortable for providers? Why do you think they are the most comfortable? - Which sites are most comfortable for clients/people getting tested? Why do you think they are the most comfortable? |
| How do you think communities would feel about mobile-based HCT? | What about rural communities in particular? What about adolescents? What about LGBTQIA communities? |
| What are some (positive or negative) consequences you could think of that might result from mobile-based HCT? | How do you think stigma impacts the number of people that test in mobile units?  What are some ways we could reduce stigma and encourage people to test in mobile units? |
| What opportunities could there be for telehealth support in MHCT? |  |
| For what aspects of the HIV care continuum could mobile units be useful? | How do you think MHCT could be useful for linking HIV positive patients to care?  How do you think MHCT could be useful for linking HIV negative patients to prevention like PrEP, STI screenings, etc.? |
| What methods would be best for advertising and promoting mobile-based HCT? |  |
| Who else should we talk to about MHCT? |  |
| In some settings, clients who test for HIV are offered HIV self-testing kits to share with others they know who might benefit from HIV testing but are not able to come to a clinic for testing. What are your thoughts on self-tests that can be done by clients in their own homes (or anywhere) to screen for HIV? | What are the pros and cons of this type of testing?  Can you think of ways that HIV self-testing might help improve HIV testing in rural Alabama?  **Does your organization have protocols for HIV self-testing procedures and processes (such as counseling, hot-line, resources, referrals, linkage etc.?)** |
| What are your thoughts on HIV testing integrated into health screening for other disease like COVID-19 or diabetes or high blood pressure? | What are the pros and cons of this type of testing?  Do you think it would increase or decrease or have no effect on testing? |
| What other interventions might improve uptake of HIV testing, particularly in rural Alabama? |  |
| What else you would like to add before we end our discussion? |  |

- 1. 1. Wise JM, Ott C, Azuero A, et al. Barriers to HIV Testing: Patient and Provider Perspectives in the Deep South. *AIDS Behav.* Apr 2019;23(4):1062-1072.
