# Supplementary material for: Rapid qualitative analysis approach to stakeholder and client interviews to inform mobile-based HIV testing in the U.S. Deep South
Source: Arch Public Health. 2023 Feb 15;81:24. doi: 10.1186/s13690-023-01039-w (PMC9930722; doi:10.1186/s13690-023-01039-w)
Supplement: Supplementary file 2 — Additional file 2. Interview Guide for HIV Testing Clients. [file 13690_2023_1039_MOESM2_ESM.docx]

Big Data Study

Interview Guide for HIV Testing Clients

| PARTICIPANT ID: | DATE: | START TIME: | END TIME: | INTERVIEWER: |
| --- | --- | --- | --- | --- |

Reminder to interviewers:

Questions in the left-hand column will be asked of all participants, while questions in the right-hand columns are probes, and are to be asked only to help the participant describe their experience or if the participant does not bring up these topics spontaneously.

Interview script:

Hello, my name is [INTERVIEWER NAME]. I am from the University of Alabama at Birmingham (UAB). We are conducting interviews with people in Alabama who have been tested for HIV, people who are newly diagnosed with HIV, and people who have never been tested for HIV to better understand what makes it easier and harder to access testing in Alabama.

As a reminder, you are not required to answer my questions, and you may skip any questions that make you uncomfortable. If you decide that you no longer want to participate in this interview, we can end at any time. As discussed, I will use a digital recorder to record our conversation.

What questions do you have before we begin the interview?

(TURN ON DIGITAL RECORDER)

| **IDI Guide Starts Here**  I will use the word community a lot in this guide. You may think of your community as the physical environment where you live. You may also think of your community as the people who you interact with on a daily basis (maybe the people where you live and where you travel during the days). Or you might think of community as people who are like you – in terms of gender, sexual identity, race, job, etc. You can think about any of these forms of community as you answer these items. | |
| --- | --- |
| **Main Questions** | **Probes** |
| (1) Tell me about any sexual health care services where you live (e.g. for STIs or contraception or HIV prevention or treatment). | Where are these services offered?  Do you think people in your neighborhood are willing to use these services? Why or why not? What about people in your peer group?  Are you comfortable using the services at these settings? Why or why not? |
| (2) How could sexual health care services be more accessible where you live? | What are some factors that make it more difficult to use sexual health care services?  What are some factors that make it easier to use sexual health care services? |
| (3) In your peer group, what kinds of discussions are happening around HIV? | Where have you heard discussions or seen information about HIV?  How did you feel in these settings when HIV was part of the discussion? |
| (4) Do you know of any HIV testing locations in your neighborhood? Tell me a little about them. | What types of settings are these sites? (health department, hospital, private practice, mobile, sexual health clinic, community center, etc.)  Where are these HIV testing locations? Are they easy to get to?  Who uses these testing sites?  What may make it easier to get tested at these sites?  What may make it more difficult to get tested at these sites?  Tell me more… |
| (5) What do people think about HIV testing in your peer group? |  |
| (6) Concerns about the confidentiality of HIV test results and procedures of the follow-up care can motivate some people to test outside of their own neighborhood – sometimes traveling to different counties or towns. What are your thoughts about this? |  |
| (7) What can test sites or health care providers do to make it easier to get tested for HIV? | Are there specific locations or settings that might be better than others? |
| (8) Some organizations conduct mobile-based HIV testing – where the counselors load their supplies into a van and move out of the clinic and into the community to conduct testing – for example at a church or a community fair or in a shopping center parking lot.  Do you know about any mobile HIV testing in your community? | Tell me about that… |
| (9) How do you think mobile-based testing could impact HIV testing? | What are some aspects that would make it easier?  What are some aspects that would make it more difficult?  What if it was associated with other infectious disease testing, like STI or COVID-19 testing?  What if it was linked to other health care screening like high blood pressure or diabetes testing? |
| (10) Some groups have been distributing home self-testing kits for clients who test to share with other people who are friends, family, or sexual contacts.  Have you heard about any home-based HIV testing available in your community? | Tell me about that…  What are some aspects of self-testing that might make HIV testing easier?  What are some aspects that might make it more difficult?  How do you feel about people distributing tests to their friends or family? |
| (11) Scientists now know that when a person with HIV is virally suppressed – meaning the amount of virus in their body is so low it can’t be detected—then they can’t transmit the virus to anyone else through sexual contact. We call this concept U=U, standing for Undetectable = Untransmissible. ^4^  Tell me what you have heard about this, if anything. | How do you think the idea that U=U (undetectable = untransmissible) might affect the way people think about HIV? What about HIV testing?  How does this affect the way *you* think about HIV or HIV testing? |
| (12) How do you think people living with HIV in your community are treated or would be treated if people knew their HIV-status? | How do you feel about that? |
| (13) In your opinion, what are some reasons that people hesitate to take an HIV test? | How does fear of being HIV positive impact whether or not people test?  How does fear of other people knowing impact whether or not people test? |
| **If it is okay with you, I am now going to ask you some questions about your personal experiences with HIV testing. If you don’t want to answer a question, just tell me, and we will move on.**  *If participant does not want to answer questions about their experiences, skip to (21).* | |
| (14) Have you ever been tested for HIV? | Can you walk me through how you decided to test or not to test for HIV? |
| (15) What factors affect your decision to get tested or not? | How does cost impact your decision to get tested or not?  How does your risk of being infected with HIV impact your decision to get tested or not?  How does finding out the result of the test impact whether or not you get tested?  How do provider recommendations for testing impact your decision to get tested or not? |
| *Ask the following questions if the participant* ***HAS*** *been tested for HIV. If not, skip to (21).* | |
| (16) How many times have you tested for HIV? | *If tested more than once*: When was the first time you tested for HIV? When was the most recent time you tested for HIV? |
| (17) Where have you tested for HIV?  Please describe your experience testing for HIV. | What were the circumstances around you getting tested there?  Why did you choose that setting/location?  Did anyone refer or suggest you get tested?  How easy or difficult was it to get to the testing site?  How did you feel inside the testing site?  When did you decide to test for HIV?  What was going through your mind before you tested for HIV?  What concerns did you have about being tested?  What did you do after testing?  What was going through your mind after testing? |
| (18) Did you tell anyone you were going to be tested? Why or why not? | Did anyone go with you to be tested? Why or why not?  *If yes:* How did they support you while you were being tested and/or while you waited for your results?  What other kinds of support do you wish you had while you tested and/or while you waited for your results? |
| (19) Did you receive the results of your HIV test(s)? | How did you receive the results?  How long did it take to receive them?  How did you feel when you received them?  What happened after you received your results? Were you contacted by anyone? (health workers, advocates, peer supports, prevention specialist, counselor, etc.) |
| (20) Can you think of anything that would have made the testing experience better? |  |
| *Ask the following question of* ***ALL*** *participants.* | |
| (21) Can you describe what you think the process would be after testing if someone were to receive negative test results? |  |
| (22) What do you think the process is for someone who receives positive test results? |  |
| (23) How easy or difficult do you think it would be to access care for someone who tests positive? | For example, counseling services or HIV medications?  Can you think of anything that would make it easier or harder to get this care? |
| **Concluding Questions** | |
| (24) Is there anything else you can think of that might help us to better support people to get tested for HIV in Alabama? |  |
| (25) Do you have any questions about this study or anything else before we end? |  |
| We want to thank you for your time today. Your answers to our questions will help us better understand how to promote HIV testing in Alabama, support those who want to get tested, and provide quick access to quality care and support for people living with HIV. | |

References:

1. Kiplagat, J., Mwangi, A., Chasela, C., & Huschke, S. (2019). *In-Depth Interview guide: Characteristics, outcomes and experiences of HIV infected adults aged 50 years and older in western Kenya.* <https://static-content.springer.com/esm/art%3A10.1186%2Fs12889-019-7283-2/MediaObjects/12889_2019_7283_MOESM1_ESM.pdf>
2. Harichund, C., Moshabela, M., & Karim, A. (2018). *Phase 2 In-Depth Interview Guide: Acceptability and Feasibility of HIV self-testing in KwaZulu-Natal, South Africa*. <https://journals.plos.org/plosone/article/file?type=supplementary&id=info:doi/10.1371/journal.pone.0212343.s001>
3. World Health Organization. *Snap Questionnaire Client Instrument*. <https://www.who.int/hiv/pub/operational/or_generic_client.pdf>
4. RW Eisinger, CW Dieffenbach, AS Fauci. HIV viral load and transmissibility of HIV infection: undetectable equals untransmittable. Journal of the American Medical Association DOI: 10.1001/jama.2018.21167 (2019).
5. STRIVE. (2012). *Measuring HIV stigma and discrimination*. https://www.icrw.org/wp-content/uploads/2017/07/STRIVE_stigma-brief-A4.pdf
