# Supplementary material for: Rapid qualitative analysis approach to stakeholder and client interviews to inform mobile-based HIV testing in the U.S. Deep South
Source: Arch Public Health. 2023 Feb 15;81:24. doi: 10.1186/s13690-023-01039-w (PMC9930722; doi:10.1186/s13690-023-01039-w)
Supplement: Supplementary file 3 — Additional file 3. Big Data Stakeholder Interview Summary. [file 13690_2023_1039_MOESM3_ESM.docx]

**BIG DATA STAKEHOLDER INTERVIEW SUMMARY**

| **Participant ID:** | **Interview Date:** | **Interviewer:** |  | **Summarizer:** | **CFIR Determinants:**  **A – Intervention Characteristics**  **B – Outer-Setting**  **C – Inner-Setting**  **D – Characteristics of Individuals**  **E – Process** |
| --- | --- | --- | --- | --- | --- |

Table of Contents

[Big Data Questions 3](#_Toc74222401)

[Barriers and Promoters of testing 3](#_Toc74222402)

[Procedure before/during/after testing 3](#_Toc74222403)

[COVID-19 Impact 3](#_Toc74222404)

[Rurality/Poverty 3](#_Toc74222405)

[Stigma 3](#_Toc74222406)

[Race 3](#_Toc74222407)

[Community knowledge of HIV (Myths, Misconceptions, misinformation) 3](#_Toc74222408)

[Trust in Healthcare System 3](#_Toc74222409)

[Community Involvement (policy support, perspectives, stakeholder engagement) 4](#_Toc74222410)

[Messaging (not a death sentence, PrEP, U=U, others) 4](#_Toc74222411)

[How to spend funding 4](#_Toc74222412)

[Other interventions 4](#_Toc74222413)

[MOBILISE Questions 4](#_Toc74222414)

[Advantages of MHCT 4](#_Toc74222415)

[Disadvantages of MHCT 4](#_Toc74222416)

[Sites for MHCT 5](#_Toc74222417)

[Community Perceptions (implementation climate) 5](#_Toc74222418)

[Resources/Support 5](#_Toc74222419)

[Linkage to Care 5](#_Toc74222420)

[Telehealth support 5](#_Toc74222421)

[Advertising 5](#_Toc74222422)

[Self-testing kits 5](#_Toc74222423)

[Integrating into wellness screenings 5](#_Toc74222424)

| **Summary**  **(key points, notes, comments, thoughts)** |
| --- |
|  |

# Big Data Questions

| **Theme/Domain:** | Barriers and Promoters of testing | **CFIR Determinants:** | A |
| --- | --- | --- | --- |
| **Key points:** |  | | |
| **Quotes:** |  | | |
| **Intersecting Domains:** |  | | |

| **Theme/Domain:** | Procedure before/during/after testing | **CFIR Determinants:** |  |
| --- | --- | --- | --- |
| **Key points:** |  | | |
| **Quotes:** |  | | |
| **Intersecting Domains:** |  | | |

| **Theme/Domain:** | COVID-19 Impact | **CFIR Determinants:** | B |
| --- | --- | --- | --- |
| **Key points:** |  | | |
| **Quotes:** |  | | |
| **Intersecting Domains:** |  | | |

| **Theme/Domain:** | Rurality/Poverty | **CFIR Determinants:** | B, D |
| --- | --- | --- | --- |
| **Key points:** |  | | |
| **Quotes:** |  | | |
| **Intersecting Domains:** |  | | |

| **Theme/Domain:** | Stigma | **CFIR Determinants:** | B, D |
| --- | --- | --- | --- |
| **Key points:** |  | | |
| **Quotes:** |  | | |
| **Intersecting Domains:** |  | | |

| **Theme/Domain:** | Race | **CFIR Determinants:** | B |
| --- | --- | --- | --- |
| **Key points:** |  | | |
| **Quotes:** |  | | |
| **Intersecting Domains:** |  | | |

| **Theme/Domain:** | Community knowledge of HIV (Myths, Misconceptions, misinformation) | **CFIR Determinants:** | D, E |
| --- | --- | --- | --- |
| **Key points:** |  | | |
| **Quotes:** |  | | |
| **Intersecting Domains:** |  | | |

| **Theme/Domain:** | Trust in Healthcare System | **CFIR Determinants:** | B, D |
| --- | --- | --- | --- |
| **Key points:** |  | | |
| **Quotes:** |  | | |
| **Intersecting Domains:** |  | | |

| **Theme/Domain:** | Maps Feedback | **CFIR Determinants:** |  |
| --- | --- | --- | --- |
| **Key points:** |  | | |
| **Quotes:** |  | | |
| **Intersecting Domains:** |  | | |

| **Theme/Domain:** | Community Involvement (policy support, perspectives, stakeholder engagement) | **CFIR Determinants:** | C, E |
| --- | --- | --- | --- |
| **Key points:** |  | | |
| **Quotes:** |  | | |
| **Intersecting Domains:** |  | | |

| **Theme/Domain:** | Messaging (not a death sentence, PrEP, U=U, others) | **CFIR Determinants:** | E |
| --- | --- | --- | --- |
| **Key points:** |  | | |
| **Quotes:** |  | | |
| **Intersecting Domains:** |  | | |

| **Theme/Domain:** | How to spend funding | **CFIR Determinants:** | A |
| --- | --- | --- | --- |
| **Key points:** |  | | |
| **Quotes:** |  | | |
| **Intersecting Domains:** |  | | |

| **Theme/Domain:** | Other interventions | **CFIR Determinants:** | A |
| --- | --- | --- | --- |
| **Key points:** |  | | |
| **Quotes:** |  | | |
| **Intersecting Domains:** |  | | |

# MOBILISE Questions

| **Theme/Domain:** | Advantages of MHCT | **CFIR Determinants:** | A |
| --- | --- | --- | --- |
| **Key points:** |  | | |
| **Quotes:** |  | | |
| **Intersecting Domains:** |  | | |

| **Theme/Domain:** | Disadvantages of MHCT | **CFIR Determinants:** | A |
| --- | --- | --- | --- |
| **Key points:** |  | | |
| **Quotes:** |  | | |
| **Intersecting Domains:** |  | | |

| **Theme/Domain:** | Sites for MHCT | **CFIR Determinants:** | A |
| --- | --- | --- | --- |
| **Key points:** |  | | |
| **Quotes:** |  | | |
| **Intersecting Domains:** |  | | |

| **Theme/Domain:** | Community Perceptions (implementation climate) | **CFIR Determinants:** | C |
| --- | --- | --- | --- |
| **Key points:** |  | | |
| **Quotes:** |  | | |
| **Intersecting Domains:** |  | | |

| **Theme/Domain:** | Resources/Support | **CFIR Determinants:** | C |
| --- | --- | --- | --- |
| **Key points:** |  | | |
| **Quotes:** |  | | |
| **Intersecting Domains:** |  | | |

| **Theme/Domain:** | Linkage to Care | **CFIR Determinants:** |  |
| --- | --- | --- | --- |
| **Key points:** |  | | |
| **Quotes:** |  | | |
| **Intersecting Domains:** |  | | |

| **Theme/Domain:** | Telehealth support | **CFIR Determinants:** | C |
| --- | --- | --- | --- |
| **Key points:** |  | | |
| **Quotes:** |  | | |
| **Intersecting Domains:** |  | | |

| **Theme/Domain:** | Advertising | **CFIR Determinants:** |  |
| --- | --- | --- | --- |
| **Key points:** |  | | |
| **Quotes:** |  | | |
| **Intersecting Domains:** |  | | |

| **Theme/Domain:** | Self-testing kits | **CFIR Determinants:** |  |
| --- | --- | --- | --- |
| **Key points:** |  | | |
| **Quotes:** |  | | |
| **Intersecting Domains:** |  | | |

| **Theme/Domain:** | Integrating into wellness screenings | **CFIR Determinants:** | E |
| --- | --- | --- | --- |
| **Key points:** |  | | |
| **Quotes:** |  | | |
| **Intersecting Domains:** |  | | |
