# Supplementary material for: Rapid qualitative analysis approach to stakeholder and client interviews to inform mobile-based HIV testing in the U.S. Deep South
Source: Arch Public Health. 2023 Feb 15;81:24. doi: 10.1186/s13690-023-01039-w (PMC9930722; doi:10.1186/s13690-023-01039-w)
Supplement: Supplementary file 4 — Additional file 4. Big Data Client Interview Summary. [file 13690_2023_1039_MOESM4_ESM.docx]

**BIG DATA CLIENT INTERVIEW SUMMARY**

| **Participant ID:** | **Interview Date:** | **Interviewer:** |  | **Summarizer:** | **CFIR Determinants:**  **A – Intervention Characteristics**  **B – Outer-Setting**  **C – Inner-Setting**  **D – Characteristics of Individuals**  **E – Process** |
| --- | --- | --- | --- | --- | --- |

Table of Contents

[Barriers and Promoters of sexual healthcare and HIV testing (including personal reasons for testing, personal barriers to testing) 3](#_Toc84584452)

[Rurality/Poverty 3](#_Toc84584453)

[Stigma 3](#_Toc84584454)

[Race 3](#_Toc84584455)

[Community knowledge of and discussions about HIV 3](#_Toc84584456)

[Community Involvement (policy support, perspectives, stakeholder engagement) 3](#_Toc84584457)

[Mobile testing thoughts (positive, negative, etc.) 3](#_Toc84584458)

[Distribution of self-testing kits 4](#_Toc84584459)

[U=U 4](#_Toc84584460)

[Experience with testing (pre-, during, and post-testing) & hypothetical experience post-testing 4](#_Toc84584461)

[Supports (family, peer, community, etc.) 4](#_Toc84584462)

[Discussions of religion and faith-based leaders 4](#_Toc84584463)

[Target groups for testing, people who get tested, people who don’t get tested 4](#_Toc84584464)

[Suggestions for improvement 4](#_Toc84584465)

| **Summary**  **(key points, notes, comments, thoughts)** |
| --- |
|  |

| **Theme/Domain:** | Barriers and Promoters of sexual healthcare and HIV testing (including personal reasons for testing, personal barriers to testing) | **CFIR Determinants:** | A |
| --- | --- | --- | --- |
| **Key points:** |  | | |
| **Quotes:** |  | | |
| **Intersecting Domains:** |  | | |

| **Theme/Domain:** | Rurality/Poverty | **CFIR Determinants:** | C, D |
| --- | --- | --- | --- |
| **Key points:** |  | | |
| **Quotes:** |  | | |
| **Intersecting Domains:** |  | | |

| **Theme/Domain:** | Stigma | **CFIR Determinants:** | B, C, D |
| --- | --- | --- | --- |
| **Key points:** |  | | |
| **Quotes:** |  | | |
| **Intersecting Domains:** |  | | |

| **Theme/Domain:** | Race | **CFIR Determinants:** | B, D |
| --- | --- | --- | --- |
| **Key points:** |  | | |
| **Quotes:** |  | | |
| **Intersecting Domains:** |  | | |

| **Theme/Domain:** | Community knowledge of and discussions about HIV | **CFIR Determinants:** | D, E |
| --- | --- | --- | --- |
| **Key points:** |  | | |
| **Quotes:** |  | | |
| **Intersecting Domains:** |  | | |

| **Theme/Domain:** | Community Involvement (policy support, perspectives, stakeholder engagement) | **CFIR Determinants:** | C, E |
| --- | --- | --- | --- |
| **Key points:** |  | | |
| **Quotes:** |  | | |
| **Intersecting Domains:** |  | | |

| **Theme/Domain:** | Mobile testing thoughts (positive, negative, etc.) | **CFIR Determinants:** | A |
| --- | --- | --- | --- |
| **Key points:** |  | | |
| **Quotes:** |  | | |
| **Intersecting Domains:** |  | | |

| **Theme/Domain:** | Distribution of self-testing kits | **CFIR Determinants:** | A |
| --- | --- | --- | --- |
| **Key points:** |  | | |
| **Quotes:** |  | | |
| **Intersecting Domains:** |  | | |

| **Theme/Domain:** | U=U | **CFIR Determinants:** | A |
| --- | --- | --- | --- |
| **Key points:** |  | | |
| **Quotes:** |  | | |
| **Intersecting Domains:** |  | | |

| **Theme/Domain:** | Experience with testing (pre-, during, and post-testing) & hypothetical experience post-testing | **CFIR Determinants:** | A |
| --- | --- | --- | --- |
| **Key points:** |  | | |
| **Quotes:** |  | | |
| **Intersecting Domains:** |  | | |

| **Theme/Domain:** | Supports (family, peer, community, etc.) | **CFIR Determinants:** | B, C, D |
| --- | --- | --- | --- |
| **Key points:** |  | | |
| **Quotes:** |  | | |
| **Intersecting Domains:** |  | | |

| **Theme/Domain:** | Discussions of religion and faith-based leaders | **CFIR Determinants:** |  |
| --- | --- | --- | --- |
| **Key points:** |  | | |
| **Quotes:** |  | | |
| **Intersecting Domains:** |  | | |

| **Theme/Domain:** | Target groups for testing, people who get tested, people who don’t get tested | **CFIR Determinants:** | B, C, D |
| --- | --- | --- | --- |
| **Key points:** |  | | |
| **Quotes:** |  | | |
| **Intersecting Domains:** |  | | |

| **Theme/Domain:** | Suggestions for improvement | **CFIR Determinants:** | B, C, D |
| --- | --- | --- | --- |
| **Key points:** |  | | |
| **Quotes:** |  | | |
| **Intersecting Domains:** |  | | |
